# Diffuse Esophageal Hyperkeratosis: A Rare Case Presentation

**DOI:** 10.7759/cureus.38757

**Published:** 2023-05-09

**Authors:** Veer Choudhry, John Williams, Umesh Choudhry, Vivek Kaul

**Affiliations:** 1 College of Medicine, University of South Florida, Tampa, USA; 2 Pathology, Gastro Florida, Clearwater, USA; 3 Gastroenterology, Morton Plant Hospital, Clearwater, USA; 4 Gastroenterology, University of Rochester Medical Center, Rochester, USA

**Keywords:** diffuse esophageal hyperkeratosis, barrett's, leukoplakia, dysphagia, parakeratosis, gastroesophageal reflux disease (gerd), gi endoscopy, hyperkeratosis, esophageal hyperkeratosis

## Abstract

Diffuse esophageal hyperkeratosis (DEH) is a very intriguing and impressive mucosal finding that is quite easily identified on endoscopy and histology. A distinction must be made between microscopic/focal hyperkeratosis and endoscopically visible DEH. Microscopic hyperkeratosis is not uncommon in histological studies, while diffuse hyperkeratosis is seen very rarely. Over the past century, only a handful of cases have been reported.

The endoscopic appearance of hyperkeratosis is of thick, white, piled-up mucosa. On histology, there is a prominent thickening of the stratum corneum, the squamous cells are anuclear, and there is no hyperplasia of the squamous epithelium. These histological characteristics distinguish benign orthokeratotic hyperkeratosis from other premalignant entities such as parakeratosis or leukoplakia where hyperplastic squamous cells retain pyknotic nuclei, lack keratohyalin granules, and also lack complete keratinization in superficial epithelial cells. The clinical presentation of hyperkeratosis includes gastroesophageal reflux, hiatal hernia, and associated symptoms.

Our case highlights a very rare endoscopic finding associated with a common clinical presentation. The nearly 10-year follow-up reinforces the benign nature of ortho-hyperkeratosis and our report underscores the features that distinguish DEH from premalignant conditions. It merits additional research into factors that lead to hyperkeratinization of the esophageal mucosa as opposed to the more common columnar metaplasia. The concomitant presence of Barrett’s esophagus in some patients is even more intriguing. Animal models with variable pH and content of the refluxate may shed light on the role played by duodenogastric/non-acid reflux in this condition. Larger, prospective, multicenter studies may provide the answers.

## Introduction

Normal esophageal squamous mucosa has a smooth pearly pink endoscopic appearance. Hyperkeratosis is a condition where the squamous mucosa has an abnormally thickened, irregular, white, and piled-up endoscopic appearance with corresponding histological changes. It is occasionally identified microscopically [1]. Diffuse esophageal hyperkeratosis (DEH), however, is a rare occurrence with only a few cases reported in the last century [1-10]. The condition merits continued identification, reporting, and study of possible etiological factors involved that may guide management.

## Case presentation

A 60-year-old, non-smoker female with minimal alcohol intake and no significant family history of esophageal carcinoma presented to the gastrointestinal outpatient clinic in 2014. She reported having a nonproductive cough for one year with exacerbation over three months. She did not report any typical symptoms of gastroesophageal reflux but had two episodes of solid food hang-up and a “spasm-like” sensation upon drinking water. Pulmonary evaluation, barium swallow, chest X-ray, and allergy testing were all negative except for a hiatal hernia. No non-steroidal anti-inflammatory drugs (NSAIDs) or proton pump inhibitor (PPI) use was reported.

The endoscopic evaluation revealed a mixed para esophageal and sliding hiatal hernia, severe erosive esophagitis (Grade D), and columnar mucosa suspicious for Barrett’s in the distal esophagus. However, more proximally, hyperkeratotic, whitish, thick, piled-up mucosa was identified. This was initially thought to be very suspicious for a neoplastic process or leukoplakia. She was placed on twice-a-day PPI therapy. Histology specimens were negative for intestinal metaplasia distally, but proximal esophageal biopsies revealed hyperkeratotic squamous mucosa with no evidence of dysplasia or neoplasia. At four weeks, the patient reported improvement in her symptoms of cough and dysphagia. After three months of twice-daily PPI therapy, she was able to reduce to once-daily dosing. She was advised of a follow-up endoscopy, which she declined. She continued pantoprazole 40 mg daily for one year and then due to concerns about the long-term PPI therapy, switched herself to lansoprazole 15 mg over-the-counter (OTC) once at night. Her other medications included OTC ginkgo biloba as an antioxidant, as well as St. John’s wort and olive leaf extract for a sense of well-being.

**Figure 1:**
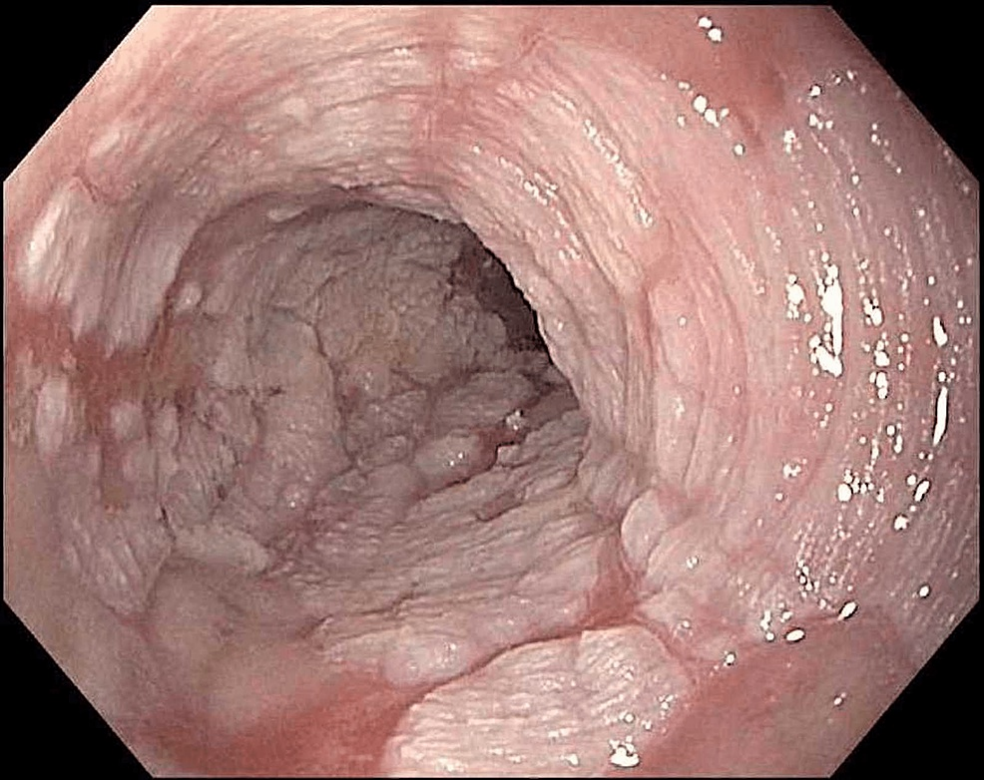
4 cm Circumferential Segment of Hyperkeratosis Between 27 and 31 cm From Incisors

She was seen again in the clinic in 2021 with the presentation of left lower quadrant pain and diverticulitis. No endoscopic workup was done between 2014 and 2021. Due to concerns about her previous endoscopic findings, a follow-up endoscopy was conducted which revealed complete healing of erosive esophagitis. Columnar-type mucosa was again identified between 31 and 35 cm from incisors. Diffuse hyperkeratosis was much more prominent and identifiable in a 4 cm circumferential segment extending between 27 and 31 cm from incisors (Figure 1). Histology again confirmed hyperkeratosis but no dysplasia or neoplasia. The proximal esophagus at 25 cm had linear furrows and histology revealed eosinophilic abundance at 60 per high-powered field (hpf). She was again placed on pantoprazole 40 mg once daily and scheduled for a follow-up endoscopy in one year.

**Figure 2:**
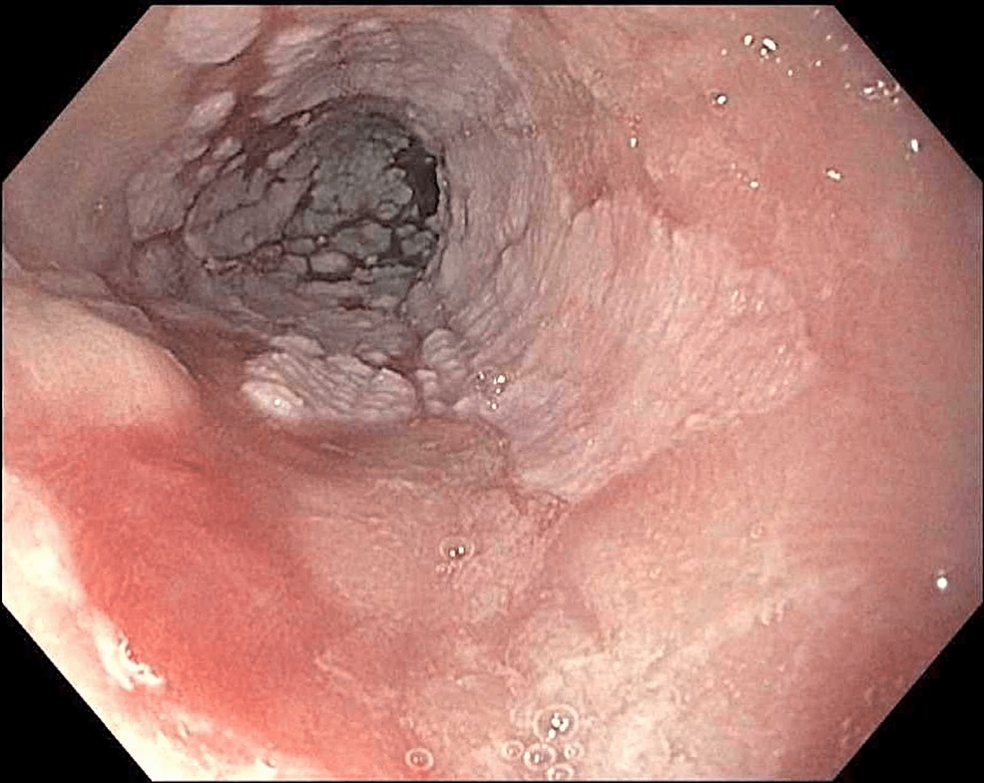
Esophageal Mucosa Proximal to Hyperkeratotic Region

**Figure 3:**
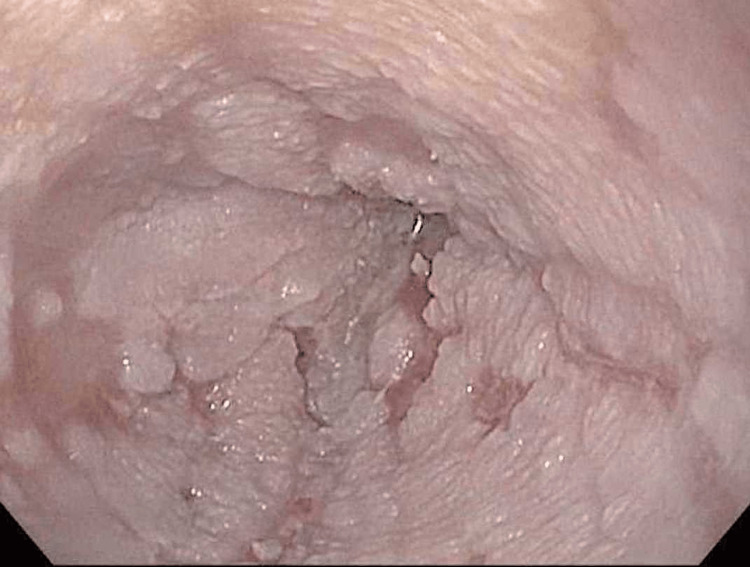
Close-up of Hyperkeratinized Esophageal Mucosa

**Figure 4:**
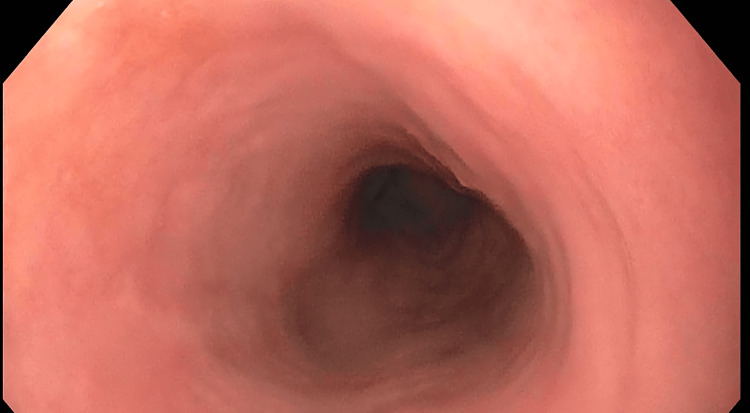
Normal Pearly Pink Esophageal Squamous Epithelium at 18 cm From Incisors

**Figure 5:**
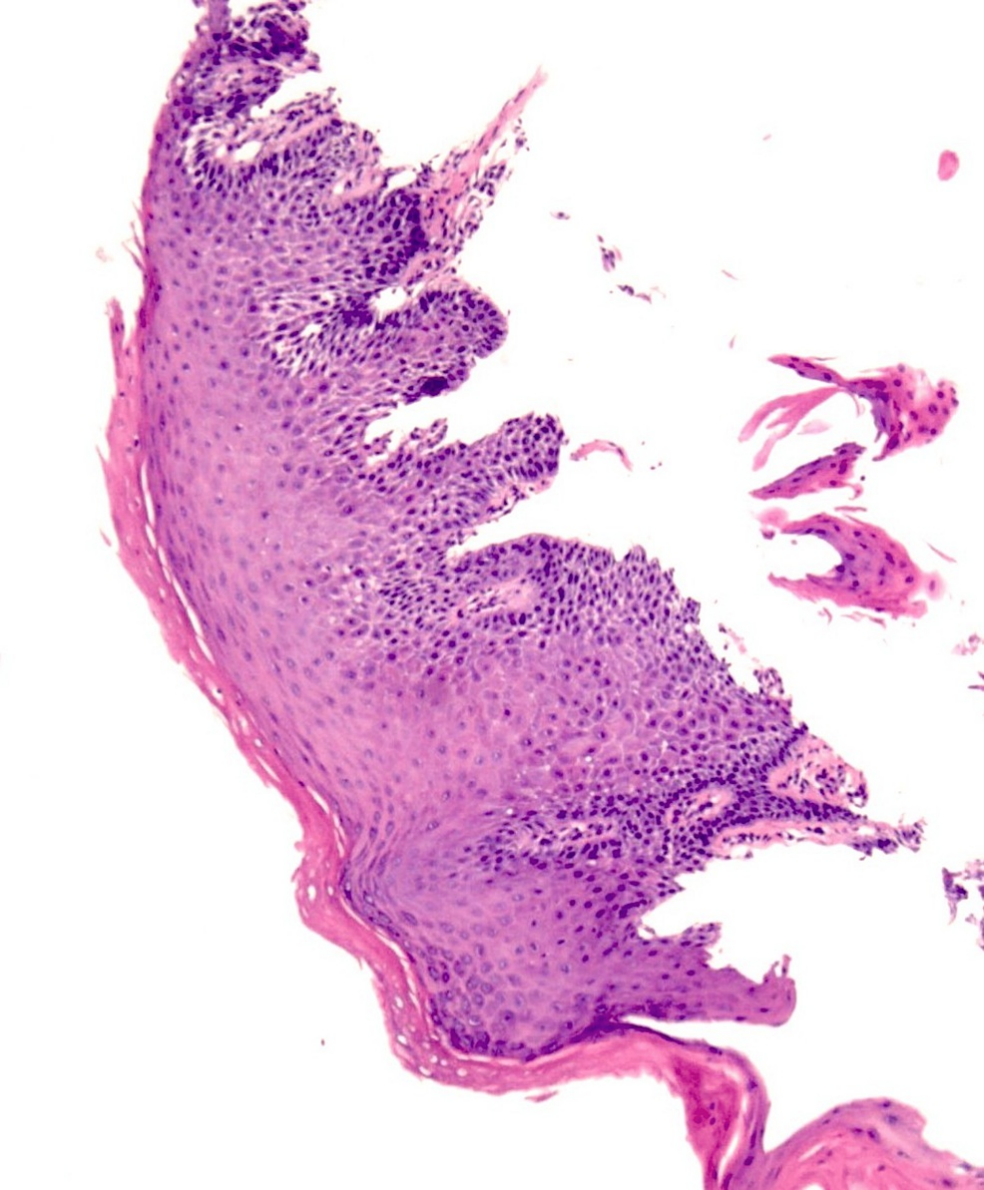
Histology of Esophageal Hyperkeratosis

The follow-up endoscopy in 2022 again showed a prominent 4 cm circumferential segment of the hyperkeratotic esophageal mucosa. Being asymptomatic, she requested a dose reduction to 20 mg of omeprazole once daily. A further follow-up and endoscopy were done at one year in February 2023. She reported slight suboptimal control of reflux but no cough. Endoscopically, the DEH persisted and was quite remarkably delineated from the remainder of the “normal-appearing mucosa”. There was no evidence of dysplasia or neoplasia in the hyperkeratotic segment. The distal columnar appearing mucosa now showed focal intestinal metaplasia. The patient's most recent endoscopy and histology images are presented (Figures 2-5) along with a review of the related literature.

## Discussion

DEH is a very intriguing and impressive mucosal finding that is quite easily identified on endoscopy and histology. The appearance is of thick, white, piled-up squamous mucosa. It is easily distinguishable from the smooth pearly pinkish normal esophageal squamous epithelium (Figure 4). The mucosa is not nodular or friable as may be seen in dysplasia or squamous carcinoma. Endoscopic features also differ from the tiny, isolated squamous mucosa seen in glycogenic acanthosis. There is no pseudomembrane formation as may be seen in esophageal candidiasis.

Histology typically reveals prominent thickening of the stratum corneum, squamous cells that are anuclear, and no hyperplasia of the squamous epithelium (Figure 5). There is an absence of a glycogen layer above the basal zone, fungal hyphae, or dysplasia. The anuclear cells without hyperplasia distinguish benign hyperkeratosis from other entities such as parakeratosis or leukoplakia where squamous cells retain pyknotic nuclei and have hyperplasia. The term hyperkeratosis is at times used interchangeably with orthokeratotic hyperkeratosis while parakeratotic hyperkeratosis is referred to simply as parakeratosis.

At the outset, a distinction must be made between microscopic hyperkeratosis and endoscopically visible DEH. Microscopic hyperkeratosis is not uncommon in histological studies. Taggart et al., in their histological study of 1845 biopsies from 98 at-risk patients, found that microscopic hyperkeratosis was present in 2% of specimens, with a great majority having three or fewer microscopic foci [1]. Visible hyperkeratosis, as reported in our patient, was identified in 0.48% of their biopsy specimens. The etiopathogenesis of DEH is not well understood. Over the past century, only a handful of cases have been reported. Starr, in 1928, reported three patients with dysphagia, luminal constriction, and histologic findings of thick, hyperkeratinized, stratified squamous epithelium resulting in a diagnosis of hyperkeratosis [2]. No determination could be made as to the cause of this condition.

Gastard et al., in 1989, further described the histology of esophageal orthokeratotic hyperkeratosis with the distinction that deeper cell layers do not display hyperplasia or dysplasia as seen with leukoplakia [3]. Additionally, the non-malignant ortho-hyperkeratosis differs from other conditions endoscopically presenting as white plaques by complete keratinization in superficial epithelial cells which are anuclear but contain keratohyalin granules. This also differs from esophageal glycogenic acanthosis, which has a layer of glycogen above the basal zone, and parakeratosis, which is associated with esophageal carcinomas. In 2005, Westerterp described a case of a 70-year-old female with a four-month history of dysphagia and endoscopic identification of a hiatal hernia along with diffuse “nearly complete keratinization of the squamous epithelium” accompanying esophageal columnar metaplasia [4]. They hypothesized that metaplastic pancreatic cells in Barrett’s esophagus of this patient led to keratinization of the mucosa. Their hypothesis was based on an earlier observation made by Pera et al. [5] in an animal model regarding the presence of pancreatic enzymes in the duodenogastric refluxate leading to differentiation in the squamous epithelium. In 2011 Kisloff et al. documented significant hyperkeratosis in a patient with a five-year history of gastroesophageal reflux disease (GERD) on a twice-daily PPI regimen [6]. The patient had “mild intraepithelial eosinophilia” without dysplasia and was negative for human papillomavirus infection (HPV) or fungal causation, which led to their conclusion that the hyperkeratosis was caused by reflux. After three consecutive one-year follow-ups, they reported the persistence of hyperkeratosis but no progression to the cancerous epithelium. This is similar to the findings in our patient after multiple follow-ups over nine years. In our patient, hyperkeratosis became more prominent initially upon the healing of the erosive esophagitis. Vitamin A deficiency and hypervitaminosis E have been shown to cause esophageal hyperkeratosis as well [7]. Ocular tests and laboratory studies ruled this out in the report by Kisloff et al. and in our patient.

In 2013, Molina-Infante et al., in a short focus report, identified linear furrows and white exudates during the endoscopic examination of a 57-year-old male with recurrent dysphagia [8]. They reported significant esophageal eosinophilic infiltration and after eight-week of treatments with rabeprazole, although eosinophilic infiltrate resolved, the esophageal squamous epithelium was noted to become hyperkeratotic with a thick granular layer but without any dysplasia. After another three months of PPI treatment, the patient was asymptomatic but hyperkeratosis persisted. All other common etiologies for hyperkeratosis such as fungal and viral infection, vitamin imbalances, cutaneous diseases, immune suppression, and genetic conditions were also ruled out. Mader et al. reported a case of an 87-year-old female with increasing severity of dysphagia but over five years of treatment, had esophageal hyperkeratosis that remained unchanged [9]. In 2009 her esophagogastroduodenoscopy (EGD) showed white plaques and keratinization in the proximal esophagus as well as intraepithelial eosinophils and a hiatal hernia. After omeprazole treatment from 2009 to 2012, her symptoms resolved but dysphagia recurred upon discontinuation of treatment. Two years later, she continued to reveal hyperkeratotic plaques. Therefore, they suggested that although DEH is a rare condition, its causal connection to GERD and dysphagia should consistently be considered. Most recently, in 2022 Sánchez-Delgado et al., in a letter to the editor, reported the case of a patient with long-term GERD, six months worsening of dysphagia, and food bolus impaction [10]. Using an old term, they described white "crackleware" squamous epithelium in the middle and lower third of the esophagus as well as LA Grade B reflux esophagitis. Biopsies showed diffuse hyperkeratosis with acanthosis and papillomatosis, but no dysplasia or carcinoma. Aggressive PPI treatment alleviated symptoms.

The case presented by us has some common features with the above reports. Namely a 4 cm hiatal hernia, Barrett’s esophagus upon confirmation of healing, and the presence of dense eosinophilic infiltration proximal to the 4 cm circumferential segment of diffuse hyperkeratosis. Viral, fungal, and immune suppression etiologies were ruled out. We agree with the thought that acid, or more likely, non-acid reflux may be the predominant etiology.

However, the question remains as to why on rare occasions a patient develops diffuse, endoscopically visible squamous hyperkeratosis instead of the usual columnar metaplasia. More confounding is the presence of both hyperkeratosis and intestinal metaplasia in the same patient as was in our patient and also reported by Taggart et al. [1]. When occurring concomitantly, hyperkeratosis is reported proximal to the intestinal metaplasia. The nine-year follow-up of our patient reinforces the benign nature of ortho-hyperkeratosis, thus making a distinction between this and parakeratosis and/or leukoplakia. Do hiatal hernia and increased contact with non-acid duodenogastric contents play a role? Other conditions where hyperkeratosis may be seen include severe esophageal dysmotility as seen in achalasia which involves no acid reflux but increased contact time with ingested food material. These conditions endoscopically often have a thick layer of adherent food material leading to a white plaque. Leukoplakia and squamous carcinoma are more common in these patients.

The role played by other, non-prescribed herbal agents including St. John's wort and ginkgo biloba, which are believed to lower esophageal sphincter pressure, is also unclear. These agents also interfere with the effectiveness of PPIs [11]. Other obvious concerns are regarding the incidence in the community, age, gender, and demographic factors. These would require a much larger sample, prospective studies, or multicenter retrospective studies.

## Conclusions

This case highlights a very rare endoscopic finding associated with a common clinical presentation, which is important from the identification standpoint of endoscopists and pathologists. Our report also aims to highlight the clinical, endoscopic, and histological differences between DEH and premalignant conditions namely leukoplakia and parakeratosis. It merits additional research into factors that lead to hyperkeratinization of the esophageal mucosa as opposed to the more commonly seen columnar metaplasia. The concomitant presence of Barrett’s esophagus in some patients is even more intriguing. Animal models with variable pH and content of the refluxate may shed light on the role played by duodenogastric/non-acid reflux in this condition. Larger, prospective, multicenter studies may provide answers and better management opportunities.
